# An Outrage on a Medical Editor

**Published:** 1899-01

**Authors:** 


### An Outrage on a Medical Editor.

"Several days ago a package, addressed to the *American Journal of Dermatology and Genito-Urinary Diseaes,* sent from Paris, was received at the St. Louis postoffice. The *Journal* was notified of its arrival and requested to send for it. Dr. S. C. Martin, associate editor of the *Journal,* called and signed for the package without knowing what it was or from whom it came. He was requested to open it, which he did, and it turned out to be books for review, which Doctor Martin was not even allowed to examine. The postoffice authorities kept them, and had Doctor Martin arrested for receiving obscene books. He was compelled to give bond and employ a lawyer to look after his interests in the case. The newspaper reporters got hold of the affair and, as usual, distorted facts for sensational effect, and under flaming headlines it was published in nearly every paper in the city that Doctor Martin had been arrested for '*sending*' obscene matter through the mails. If this action by the postoffice authorities is endorsed by the department, the editor of any medical journal is liable to arrest when he signs for any package addressed to his journal. The question arises: has a citizen any rights that federal authority is bound to respect?

Then Prosecuting Attorney Rozier, who up to this time had remained a passive spectator of the proceeding, stepped in and distangled the tangle by asking for a dismissal of the charge on the grounds that "there was nothing in it and never had been anything in it."—*St. Louis Medical Era,* November, 1898.

